# The efficacy and safety of febuxostat for urate lowering in gout patients ≥65 years of age

**DOI:** 10.1186/1471-2318-12-11

**Published:** 2012-03-21

**Authors:** Robert L Jackson, Barbara Hunt, Patricia A MacDonald

**Affiliations:** 1 Takeda Global Research & Development Centers, Inc, One Takeda Parkway, Deerfield, Illinois 60015, USA

## Abstract

**Background:**

The incidence of gout rises with increasing age. Management of elderly (≥65 years) gout patients can be challenging due to high rates of comorbidities, such as renal impairment and cardiovascular disease, and concomitant medication use. However, there is little data specifically addressing the efficacy and safety of available urate-lowering therapies (ULT) in the elderly. The objective of this post hoc analysis was to examine the efficacy and safety of ULT with febuxostat or allopurinol in a subset of elderly subjects enrolled in the CONFIRMS trial.

**Methods:**

Hyperuricemic (serum urate [sUA] levels ≥ 8.0 mg/dL) gout subjects were enrolled in the 6-month, double-blind, randomized, comparative CONFIRMS trial and randomized, 1:1:1, to receive febuxostat, 40 mg or 80 mg, or allopurinol (200 mg or 300 mg based on renal function) once daily. Flare prophylaxis was provided throughout the study duration.

Study endpoints were the percent of elderly subjects with sUA <6.0 mg/dL at the final visit, overall and by renal function status, percent change in sUA from baseline to final visit, flare rates, and rates of adverse events (AEs).

**Results:**

Of 2,269 subjects enrolled, 374 were elderly. Febuxostat 80 mg was significantly more efficacious (82.0%) than febuxostat 40 mg (61.7%; *p *< 0.001) or allopurinol (47.3%; *p *< 0.001) for achieving the primary efficacy endpoint. Febuxostat 40 mg was also superior to allopurinol in this population (*p *= 0.029). In subjects with mild-to-moderate renal impairment, significantly greater ULT efficacy was observed with febuxostat 40 mg (61.6%; *p *= 0.028) and febuxostat 80 mg (82.5%; *p *< 0.001) compared to allopurinol 200/300 mg (46.9%). Compared to allopurinol 200/300 mg, the mean percent change in sUA from baseline was significantly greater for both febuxostat 80 mg (*p *< 0.001) and febuxostat 40 mg (*p *= 0.011) groups. Flare rates declined steadily in all treatment groups. Rates of AEs were low and comparable across treatments.

**Conclusions:**

These data suggest that either dose of febuxostat is superior to commonly prescribed fixed doses of allopurinol (200/300 mg) in subjects ≥65 years of age with high rates of renal dysfunction. In addition, in this high-risk population, ULT with either drug was well tolerated.

**Trial registration:**

clinicaltrials.gov NCT#00430248

## Background

The average age of patients seeking care for gout in the United States is approximately 66 years [1]. Managing gout in the elderly (≥65 years of age) is clinically challenging as people in this demographic segment have lower creatinine clearance, a greater frequency of comorbidities, and are often taking multiple medications [2]. There is a dearth of evidence-based information on effective management of urate-lowering therapies (ULT) in elderly patients. Available ULT options in the United States include xanthine oxidase (XO) inhibitors, allopurinol, and febuxostat, as well as the uricosuric agent probenecid. Probenecid is not effective in gout patients with renal impairment [3]--30% to 60% of gout patients have some degree of renal deficiency [4]. Allopurinol, which has been the mainstay of chronic uric acid management, is traditionally dosed according to degree of renal impairment [5]. Such dose decrements often lead to reduced efficacy [6]. Febuxostat is a selective, novel, non-purine analog XO inhibitor [7] for the treatment of chronic hyperuricemia in patients with gout [8]. Primarily metabolized in the liver [9], its pharmacokinetic and pharmacodynamic profiles are not affected by age or by mild-to-moderate hepatic or renal impairment [10-12]. In this post-hoc analysis, we report the efficacy and safety of ULT with febuxostat or allopurinol in an elderly gout population.

## Methods

The CONFIRMS trial (NCT#00430248) was a 6-month, Phase 3, double-blind, randomized, controlled trial (RCT) further examining the comparative urate-lowering efficacy and safety of febuxostat and allopurinol [13]. Subjects were enrolled at 324 sites in the United States. Institutional Review Board approval was obtained, and all subjects provided written informed consent that conformed to the Declaration of Helsinki and the Health Insurance Portability and Accountability Act prior to any study-related procedure. A total of 2,269 subjects who met the American College of Rheumatology criteria for the diagnosis of gout [14] and had hyperuricemia (serum urate [sUA] ≥8.0 mg/dL [~475 μmol/L; to convert an sUA value from mg/dL to μmol/L, multiply by 59.48]) were enrolled. Study design and subject inclusion and exclusion criteria have been previously described [13]. Exclusion criteria included, but were not limited to, secondary hyperuricemia, xanthinuria, severe renal impairment (estimated creatinine clearance [eCLcr] <30 mL/min, calculated by Cockcroft-Gault formula corrected for ideal body weight[15,16]), a history of cancer within 5 years of screening, or any significant medical condition and/or conditions that would interfere with the treatment, safety, or compliance with the protocol. Subjects were randomized 1:1:1 to receive a daily dose of either febuxostat 40 mg, febuxostat 80 mg (Uloric^®^; Takeda Global Research & Development Center, Inc, Deerfield, IL), or allopurinol (Apotex, Inc, Weston, FL). Subjects randomized to allopurinol received 300 mg daily if baseline renal function was normal (eCLcr ≥90 mL/min) or mildly impaired (eCLcr 60 to <90 mL/min); subjects with moderate renal impairment (eCLcr 30 to <60 mL/min) received 200 mg daily.

Throughout the 6-month treatment period, subjects received prophylaxis for gout flares, with either colchicine (Westward Pharmaceutical Corporation, Eatontown, NJ), 0.6 mg daily, or naproxen (Westward Pharmaceutical Corporation, Eatontown, NJ), 250 mg twice daily. Subjects with eCLcr <50 mL/min were not given naproxen. All subjects receiving naproxen prophylaxis also received lansoprazole 15 mg daily (Takeda Global Research & Development Center, Inc, Deerfield, IL). If subjects could not tolerate either colchicine or naproxen, they could be prescribed other medications for flare prophylaxis.

The primary efficacy endpoint of CONFIRMS was the proportion of subjects who achieved a target level of sUA <6.0 mg/dL (~360 μmol/L) at final visit. Additional efficacy endpoints included the proportion of subjects with mild or moderate renal impairment with a final sUA <6.0 mg/dL and percent change in sUA from baseline to final visit in each treatment group. Self-reported acute gout flares requiring treatment and adverse events (AEs) were collected throughout the study; flares were not considered AEs. Flares were defined as acute episodes of joint swelling, pain, exquisite tenderness and warmth, and redness at the joint, along with the overlying skin being tense, warm, shiny, and purplish or red in color. Clinical laboratory tests were performed every 2 months. All AEs were coded using Medical Dictionary for Regulatory Activities terminology. Flare rates were summarized by 2-month periods. Our current analyses assessed the primary and secondary efficacy endpoints and safety among subjects ≥65 years of age (N = 374).

Statistical analyses of the efficacy endpoints and AE rates have been previously described in detail [13]. In the primary CONFIRMS analysis of the total cohort (N = 2,269), non-inferiority of febuxostat 40 mg compared to allopurinol was demonstrated. Binomial 95% confidence intervals (CIs) were calculated for the difference between the 2 groups in achieving the primary efficacy endpoint. Non-inferiority was declared if the lower limit of the 95% CI for difference in the proportion of subjects achieving an sUA <6.0 mg/dL at the final visit was greater than −10%. The difference between the febuxostat 40 mg group (45.2%) and the allopurinol group (42.1%) was 3.1% (95% CI:-1.9%-8.1%), thus demonstrating non-inferiority [13]. Last observation carried forward (LOCF) was used to account for missing data in the primary analysis. For subjects who prematurely discontinued the study before the Month 6 visit, the sUA value obtained at the last completed visit was used as the final visit. For the primary endpoint, pairwise comparisons were made between treatment groups shown to be non-inferior with Fisher's exact test. Statistical significance of mean percent change from baseline between treatment groups was determined using ANOVA. All primary and secondary efficacy analyses were performed on the intent-to-treat (ITT) population, which was defined as all randomized subjects who had a baseline sUA ≥8.0 mg/dL and received at least 1 dose of study drug. Safety analysis was carried out on all randomized subjects who received at least 1 dose of study drug.

## Results

Three hundred seventy-four subjects ≥65 years of age who met all of the inclusion and none of the exclusion criteria were enrolled in the CONFIRMS study. Of these, 115, 128, and 131 were randomized to receive daily febuxostat 40 mg, febuxostat 80 mg, or allopurinol 200/300 mg, respectively. Among those randomized to receive allopurinol, 80/131 (61.1%) subjects had moderate renal impairment and therefore received 200 mg daily.

**Figure 1:**
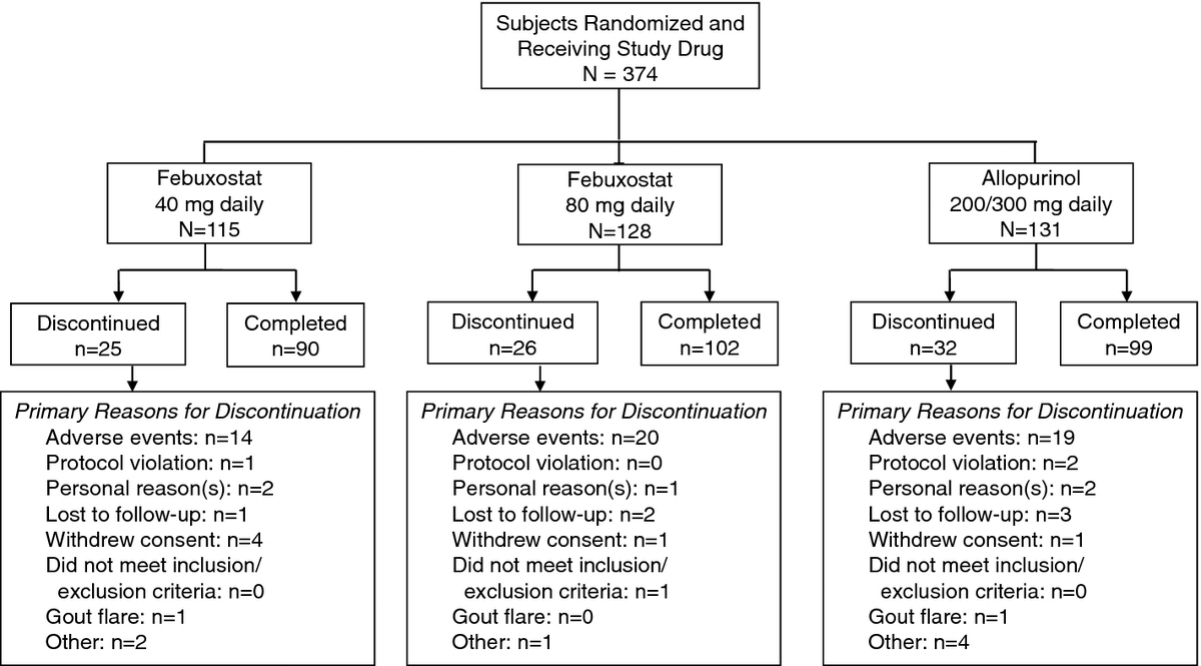
**Flow of Subjects Through the Study**.

Figure 1 illustrates the flow of subjects ≥65 years of age through the study. Of the 374 subjects, 83 (22.2%) discontinued prematurely; 28 (7.5%) discontinuations occurred during the first 30 days. Premature discontinuation rates were similar across treatment groups. The primary reason for premature discontinuation was AEs (n = 53; 14.2%).

Demographics, baseline characteristics, and medical histories were similar across treatment groups (Table 1). Subjects were predominantly male (85.8%), Caucasian (84.8%), and obese (51.3%; body mass index ≥30 kg/m^2^), with a mean age of 71 years. Mean duration of gout was 14.6 years and mean baseline sUA was 9.4 mg/dL. Tophi and kidney stones were present in 19.3% and 19.8% of subjects, respectively. Mild or moderate renal impairment was determined in 139 (37.2%) and 229 (61.2%) subjects, respectively. Comorbidities were common; in addition to the predominance of renal impairment, the majority of subjects (87.2%) had some history of cardiovascular disease, inclusive of, but not limited to, hypertension (82.4%), coronary artery disease (24.3%), cardiac arrhythmia (21.1%), and myocardial infarction (10.7%). In addition, 59.6% of elderly subjects had a history of hyperlipidemia and 24.6% were diabetic.

**Table 1:** Demographics, baseline characteristics, and comorbidities^a^

Variable	Febuxostat 40 mg N = 115	Febuxostat 80 mg N = 128	Allopurinol 200/300 mg N = 131
**Gender**, n (%)			
Male	104 (90.4)	109 (85.2)	108 (82.4)
Female	11 (9.6)	19 (14.8)	23 (17.6)

**Race**, n (%)			

American Indian or Alaska Native	1 (0.9)	1 (0.8)	0
Asian	2 (1.7)	4 (3.1)	5 (3.8)
Black or African American	10 (8.7)	16 (12.5)	8 (6.1)
Native Hawaiian or Other Pacific Islander	1 (0.9)	2 (1.6)	0
White	98 (85.2)	103 (80.5)	116 (88.5)
Other	3 (2.6)	2 (1.6)	2 (1.5)

**Ethnicity**, n (%)			
Hispanic or Latino	2 (1.7)	3 (2.3)	9 (6.9)
Not Hispanic or Latino	113 (98.3)	125 (97.7)	122 (93.1)

**Age (years)**			
Mean ± SD	70.8 ± 5.19	71.2 ± 5.22	70.1 ± 4.59
Range	65-85	65-85	65-85

**Body mass index (kg/m^2^)**			
Mean ± SD	31.0 ± 5.47	30.7 ± 5.13	31.6 ± 5.60
Range	20-48	16-49	22-48

**Alcohol use**, n (%)			
Non-/Ex-drinker	42 (36.5)	49 (38.3)	53 (40.5)
Drinker (1-14 drinks/week)	73 (63.5)	79 (61.7)	78 (59.5)

**Serum urate (mg/dL)**			
Mean ± SD	9.4 ± 1.14	9.5 ± 1.17	9.3 ± 1.04
Range	8-14	8-13	8-13

**Years with gout**			
Mean ± SD	15.0 ± 11.3	14.8 ± 12.40	14.1 ± 12.44
Range	0-53	0-50	0-48

**Tophi present**, n (%)			
No	93 (80.9)	104 (81.3)	105 (80.2)
Yes	22 (19.1)	24 (18.8)	26 (19.8)

**Participated in a previous febuxostat study^b^**, n (%)			
Yes	18 (15.7)	23 (18.0)	21 (16.0)

**Prior urate lowering therapies**, n (%)			
No	31 (27.0)	34 (26.6)	44 (33.6)
Yes (any)	84 (73.0)	94 (73.4)	87 (66.4)
Febuxostat	17 (14.8)	21 (16.4)	18 (13.7)
Allopurinol	76 (66.1)	85 (66.4)	75 (57.3)
Probenecid	5 (4.3)	6 (4.7)	6 (4.6)
Other	4 (3.5)	4 (3.1)	3 (2.3)

**Renal Function^c^**, n (%)			
Moderately Impaired	67 (58.3)	82 (64.1)	80 (61.1)
Mildly Impaired	45 (39.1)	44 (34.4)	50 (38.2)
Normal	3 (2.6)	2 (1.6)	1 (0.8)

**Medical History**, n (%)			
Any cardiovascular disease^d^	102 (88.7)	108 (84.4)	116 (88.5)
Hypertension	98 (85.2)	102 (79.7)	108 (82.4)
Coronary artery disease	26 (22.6)	28 (21.9)	37 (28.2)

Cardiac arrhythmia	16 (13.9)	28 (21.9)	35 (26.7)
Diabetes	23 (20.0)	35 (27.3)	34 (26.0)
Hypercholesterolemia	15 (13.0)	10 (7.8)	17 (13.0)
Hyperlipidemia	66 (57.4)	73 (57.0)	84 (64.1)
Use of low-dose aspirin (≤325 mg daily)	48 (41.7)	52 (40.6)	49 (37.4)

^a^There were no statistically significant differences among treatment groups with respect to the distribution of baseline characteristics.

^b^Subjects previously enrolled in prior febuxostat clinical trials were permitted to enroll in the CONFIRMS trial.

^c^Moderate baseline renal impairment: estimated creatinine clearance (eCLcr) 30- < 60 mL/min; mild baseline renal impairment: eCLcr 60- < 90 mL/min; normal: eCLcr ≥90 mL/min.

^d^ Any cardiovascular disease includes myocardial infarction, percutaneous transluminal coronary angioplasty, coronary artery bypass graft procedure, coronary artery disease, transient ischemic attack, peripheral vascular disease, cardiac arrhythmia, venous thrombotic event, valvular heart disease, congestive heart disease, and hypertension. SD = Standard deviation

Concomitant medication use was high in this population and comparable across treatment groups (Table 2). Sixty percent of elderly subjects were taking at least one medication that acted on the renin-angiotensin system, 50% were taking an antithrombotic agent, while 24% were taking at least 1 diuretic. Colchicine was the primary gout flare prophylaxis used by elderly subjects (83.7%), while 10.4% received naproxen. The remaining 5.9% of subjects received indomethacin, nabumetone, prednisone, or celecoxib as flare prophylaxis.

**Table 2:** Selected concomitant medication use among elderly subjects during the CONFIRMS trial

	Febuxostat 40 mg N = 115	Febuxostat 80 mg N = 128	Allopurinol 200/300 mg N = 131
		**n (%)**	

**Subjects with one or more concomitant medication**			

Agents acting on the renin-angiotensin system^a^	65 (57)	78 (61)	83 (63)

Analgesics	23 (20)	33 (26)	27 (21)

Antiinflammatory and antirheumatic products	26 (23)	32 (25)	26 (20)

Antithrombotic agents	58 (50)	65 (51)	65 (50)

Beta blockers	45 (39)	43 (34)	55 (42)

Calcium channel blockers	19 (17)	21 (16)	30 (23)

Corticosteroids for systemic use	12 (10)	21 (16)	15 (11)

Drugs for acid-related gastrointestinal disorders	23 (20)	32 (25)	34 (26)

Drugs used in diabetes	20 (17)	25 (20)	29 (22)

Diuretics	26 (23)	32 (25)	33 (25)

Lipid modifying agents	64 (56)	69 (54)	75 (57)

Other antihypertensives^b^	13 (11)	4 (3)	10 (8)

^a^Agents acting on the renin-angiotensin system include a direct renin inhibitor, angiotensin II receptor blockers, and angiotensin-converting enzyme inhibitors.

^b^Includes vasodilators, alpha-blockers, and α-adrenergic receptor agonists.

**Figure 2:**
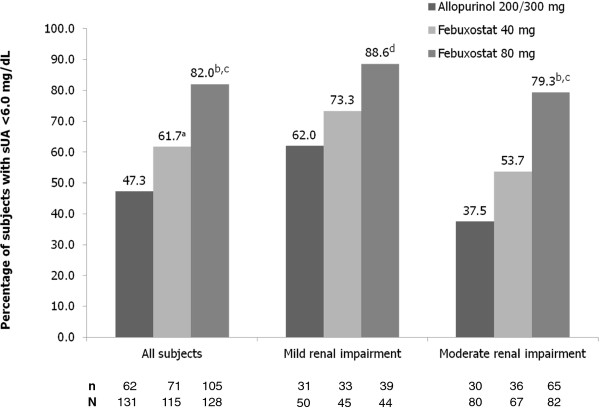
**Achievement of sUA <6.0 mg/dL--All Subjects (Primary Endpoint) and by Renal Function**. Data regarding the percentage of subjects with normal renal function with sUA <6.0 mg/dL is not presented due to the low number of subjects in this group (N = 6). ^a^*p *= 0.029 vs allopurinol; ^b^*p *≤ 0.001 vs febuxostat 40 mg; ^c^*p *< 0.001 vs allopurinol; ^d^*p *= 0.004 vs allopurinol.

Achievement of the primary efficacy endpoint, sUA <6.0 mg/dL at final visit, occurred in 47.3%, 61.7%, and 82.0% of subjects in the allopurinol 200/300 mg, febuxostat 40 mg, and febuxostat 80 mg groups, respectively (Figure 2). Febuxostat 80 mg was significantly more efficacious than febuxostat 40 mg or allopurinol for achieving the primary efficacy endpoint (*p *< 0.001 for both comparisons). In addition, febuxostat 40 mg was superior to allopurinol in this elderly gout population (*p *= 0.029).

In subjects with any renal impairment (mild or moderate), the urate-lowering efficacies (sUA of <6.0 mg/dL at final visit) of both febuxostat 40 mg (69/112; 61.6%) and febuxostat 80 mg (104/126; 82.5%) were significantly better than that of allopurinol 200/300 mg (61/130; 46.9%; *p *= 0.028 vs febuxostat 40 mg; *p *< 0.001 vs febuxostat 80 mg). Achievement of sUA <6.0 mg/dL by renal status is also illustrated in Figure 2. Febuxostat 80 mg was significantly more efficacious than both febuxostat 40 mg and allopurinol in subjects with moderate renal impairment (*p *≤ 0.001) and superior to allopurinol in subjects with mild renal impairment (*p *= 0.004). Within the mild or within the moderate renal impairment categories the percentage of subjects achieving final visit sUA <6.0 mg/dL was numerically greater in the febuxostat 40 mg group than in the allopurinol group, but the differences were not statistically significant.

**Figure 3:**
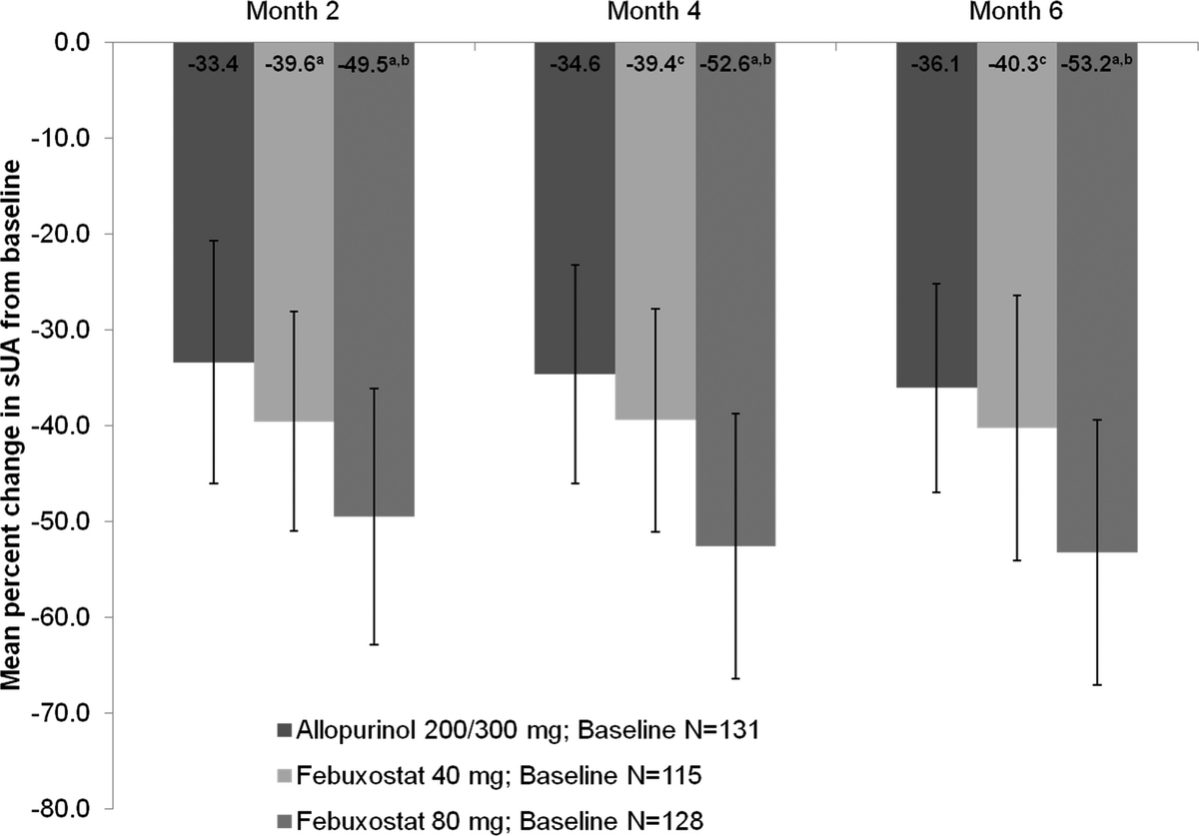
**Mean Percent Change From Baseline In Serum Urate at Each Scheduled Visit**. ^a^*p *< 0.001 vs allopurinol; ^b^*p *< 0.001 vs febuxostat 40 mg; ^c^*p *≤ 0.027 vs allopurinol. Error bars represent standard deviation.

The mean percent change (±standard deviation) in sUA from baseline to the final visit for the febuxostat 40 mg, febuxostat 80 mg, and allopurinol 200/300 mg groups was -36.9% (±16.8), -48.1% (±20.0), and -31.1% (±16.0), respectively. The mean percent change in sUA from baseline at months 2, 4, and 6 are illustrated in Figure 3. These percent changes were significantly greater in the febuxostat 80 mg group compared with either the febuxostat 40 mg or allopurinol 200/300 mg groups (*p *< 0.001 for both comparisons) and also in the febuxostat 40 mg group compared with the allopurinol 200/300 mg group (*p *= 0.011).

The proportion of subjects requiring treatment for gout flares declined in all treatment groups during the course of the study. In the febuxostat 40 mg and 80 mg treatment groups, flare rates during the first 2 months were 16.5% (19/115) and 18.0% (23/128), respectively. During the last 2 months of treatment, rates were 9.5% (9/95) and 6.6% (7/106), respectively. Treatment for gout flares was required by 4.6% (6/131) of subjects in the allopurinol 200/300 mg group during the first 2 months; this rate decreased to 0.9% (1/106) during the last 2 months. Flare rates were significantly higher in the febuxostat 40 mg and 80 mg groups compared with the allopurinol group (*p *≤ 0.05) during the first 2 months of the study, and in the febuxostat 40 mg group compared with the allopurinol group (*p *≤ 0.05) during the last 2 months.

Overall, rates of AEs were low and similar across treatment groups; the most frequently reported AEs were diarrhea (9.6%), upper respiratory tract infection (7.8%), and musculoskeletal and connective tissue signs and symptoms (6.1%) (Table 3). Rates of abnormal liver function analyses were reported in 4.3%, 3.9%, and 2.3% of subjects in the febuxostat 40 mg, febuxostat 80 mg, and allopurinol 200/300 mg groups, respectively. The percentage of subjects who discontinued prematurely from the study due to abnormal liver function analyses was 0.9%, 1.6%, and 0 in the febuxostat 40 mg, febuxostat 80 mg, and allopurinol groups, respectively. Elevated liver function tests are listed by treatment group in Table 3. No subjects experienced concurrent elevated ALT/bilirubin, AST/bilirubin, ALT/alkaline phosphatase, AST/alkaline phosphatase, or ALT/AST/bilirubin levels. Serious AEs occurred in 7.8%, 6.3%, and 11.5% of subjects in the febuxostat 40 mg, febuxostat 80 mg, and allopurinol 200/300 mg groups, respectively. Cardiac disorders and infections were the most frequently reported serious AEs; in the febuxostat 40 mg, febuxostat 80 mg, and allopurinol groups, 0.9%, 1.6%, and 3.1% of subjects reported cardiac disorders and 1.7%, 0.8%, and 3.1% reported infections and infestations. Two subjects in this elderly population died during the study; both were in the allopurinol group. One subject died from hypertensive heart disease, and the other from postsurgical complications related to lung adenocarcinoma and lymphocytic leukemia.

**Table 3:** Most frequently reported adverse events^a ^and elevated serum liver function tests, by treatment group

	Febuxostat 40 mg N = 115	Febuxostat 80 mg N = 128	Allopurinol 200/300 mg N = 131
**Total subjects reporting **≥**1 AE**, n (%)	n (%)70 (60.9)	75 (58.6)	81 (61.8)

**Most frequently reported^b ^AEs**, n (%)			

Diarrhea (non infectious)	14 (12.2)	12 (9.4)	10 (7.6)

Musculoskeletal and connective tissue signs and symptoms	10 (8.7)	7 (5.5)	6 (4.6)

Upper respiratory tract infections	9 (7.8)	11 (8.6)	9 (6.9)

Neurological signs and symptoms	8 (7.0)	1 (0.8)	1 (0.8)
(Dizziness)	7 (6.1)	1 (0.8)	1 (0.8)
(Presyncope)	1 (0.9)	0	0

Joint related signs and symptoms	2 (1.7)	8 (6.3)	3 (2.3)

Lower respiratory tract and lung infections	2 (1.7)	3 (2.3)	7 (5.3)

Headaches	6 (5.2)	2 (1.6)	3 (2.3)

Nausea and vomiting	6 (5.2)	1 (0.8)	4 (3.1)

**Elevated liver function tests**, n/N (%)			

ALT			
≥2X ULN	4/106 (4)	4/117 (3)	3/115 (3)
≥3X ULN	2/106 (2)	0/117	1/115 (<1)

AST			
≥2X ULN	3/106 (3)	1/117 (<1)	0/115
≥3X ULN	2/106 (2)	0/117	0/115

ALT and AST concurrently			
≥3X ULN	2/106 (2)	0/117	0/115

^a^AEs are reported by Medical Dictionary for Regulatory Activities high-level term.

^b^Most frequently reported AEs are those which occurred in ≥5% of elderly subjects in any treatment group.

AE = adverse event; ALT = alanine aminotransferase; AST = aspartate aminotransferase; ULN = upper limit of normal.

## Discussion

This is the first analysis of randomized, controlled clinical trial data to specifically examine the efficacy and safety of ULT with febuxostat or allopurinol in a population of elderly gout subjects with significant proportions of renal impairment and other comorbidities. In this cohort, ULT with either dose of febuxostat (40 mg or 80 mg) was significantly more efficacious than fixed doses of allopurinol 200/300 mg in achieving the therapeutic sUA goal (<6.0 mg/dL). In the total CONFIRMS study population, the efficacy of febuxostat 40 mg was comparable to that of allopurinol [13]. In this analysis, the greater efficacy of febuxostat 40 mg over allopurinol 200/300 mg can be attributed to the very high proportion of elderly patients with mild or moderate renal impairment.

Flare rates declined markedly in this group, although they were persistently higher in either febuxostat group compared with the allopurinol group. This may reflect more rapid, profound urate lowering in the febuxostat groups. The paradoxical relationship between the extent of sUA reduction and increasing flares upon initial ULT is well recognized [17,18]; rapid lowering of sUA leads to mobilization of monosodium urate crystals from the body's urate pools, thus triggering an inflammatory response [19]. It has also been demonstrated that the continued use of ULT for up to 5 years leads to near elimination of gout flares [20,21].

ULT with either febuxostat or allopurinol was well tolerated in this population, with AE rates similar to those reported for the entire CONFIRMS cohort [13]. Importantly, despite the high percentage of elderly subjects with cardiovascular disease, the rates of cardiovascular events were low and comparable across treatment groups. This reflects the lack of any significant differences in adjudicated cardiovascular AEs as previously reported across treatment groups for the full CONFIRMS population [13].

As the prevalence of gout among the elderly continues to rise [22-24], optimal management of chronic gout and its underlying hyperuricemia is necessary to improve clinical outcomes and reduce healthcare utilization and associated costs. Achievement and long-term maintenance of sUA <6.0 mg/dL leads to reduction of tophi and near-elimination of acute flares [20,21,25-27], and may stabilize or improve renal function in gout patients [28].

Gout has been identified as an independent risk factor for diabetes, cardiovascular disease, and all-cause and cardiovascular-related mortality [29-34]. While ULT has not been approved for the management of asymptomatic hyperuricemia, recent studies have suggested the cardiovascular-protective impact of lowering sUA levels [35], thereby providing potential additional clinical benefit to properly managed gout patients.

Elderly patients have a high incidence of components of the metabolic syndrome (hypertension, obesity, dyslipidemia, insulin resistance) and declining renal function, which may impair handling of the uric acid load. This, combined with the associated concomitant medication use, which impacts both renal function and uric acid [36], may further increase hyperuricemia. As a result, elderly gout patients are more challenging to manage. Recently, Keenan et al. [37] have documented high rates of inappropriate prescribing patterns of gout medications related to contraindicated concomitant medications for other comorbidities. Even cardioprotective use of low-dose aspirin (75-325 mg/day) can impair renal function and uric acid handling in elderly patients [38]. In elderly patients on thiazide diuretics, which increase the net reabsorption of uric acid in the proximal tubule of the nephron [39], the adjusted relative risk for the onset of gout, as documented by initiation of anti-gout therapy, was 1.99 (95% CI 1.21-3.26) [40]. Gout management can be further complicated by low therapeutic compliance; older gout patients have suboptimal adherence to ULT compared with therapies for other chronic illnesses [41].

Retrospective analyses of managed care databases reveal that elderly gout patients have higher all-cause healthcare utilization and costs compared with patients without gout, and this rises with increasing sUA [36,42,43]. Comparison of managed care database claims of 11,935 gout patients ≥65 years of age and matched non-gout patients found that the adjusted difference in total 12-month all-cause healthcare costs was $3,038 (*p *< 0.001; 2005 USD) [42]. Gout-related costs represent approximately 6% of total healthcare costs for elderly gout patients [42]. Along with the expected higher rate of gout-related medication use, Hanly et al. [36] reported that compared with age- and sex-matched controls, elderly gout patients had significantly higher (*p *< 0.0001) utilization rates of diuretics and insulin, which can increase sUA. Higher utilization rates (*p *< 0.0001) were also reported for beta-blockers, angiotensin-converting enzyme inhibitors, angiotensin II receptor blockers, and calcium channel blockers, a reflection of higher comorbidity rates in the gout cohort [36]. In addition, higher rates (*p *< 0.0001) of use of gastroprotective agents were noted, likely due to the higher NSAID usage for treatment of flares [36]. Additional database analyses of 2,237 elderly gout patients have shown that among those with higher sUA (>9.0 mg/dL), compared to patients with well-controlled sUA (<6.0 mg/dL), there was a higher frequency of flares, and significantly higher gout-related ($620 vs $264 per flare; *p *< 0.0001) and total healthcare costs ($4,944 vs $2,389 per flare; *p *< 0.0001) [43].

This study is not without limitations, the first of which is its post hoc nature. Even so, the data presented here are the first gathered from an RCT and provide evidence for the use of febuxostat as an option for the management of chronic grout in the elderly, particularly those with mild or moderate renal impairment. A second limitation of this analysis is the use of commonly prescribed [44,45] fixed doses of allopurinol determined by renal function. Guideline recommendations [46] to start with a low dose of allopurinol and slowly titrate up are based on an outdated, single, noncomparative study of 78 patients with renal insufficiency who all developed severe allopurinol hypersensitivity [5]. Failure to achieve target sUA with guideline-recommended doses of allopurinol has been documented [6]. In 2 recent small trials [47,48], a total of 81 gout patients, including those with mild or moderate renal impairment, received increasing allopurinol doses above creatinine clearance-based dosing guidelines, which led to improved urate-lowering efficacy without increased toxicity. Due to the very limited data regarding the safety of high doses of allopurinol in patients with renal insufficiency, allopurinol dosing in this population remains controversial [49], and we cannot speculate whether allopurinol dose titration in the CONFIRMS study, and more specifically among this elderly subgroup, would have led to improved efficacy without increased AEs. Additional studies addressing the safety of allopurinol dose titration with much greater numbers of subjects with renal impairment are needed. The doses of allopurinol used in the CONFIRMS trial reflect dosing commonly utilized in clinical practice [44,45]; therefore, the data presented here can be applied to real-world management of elderly gout patients.

Optimal management can lead to relief from the clinical manifestations of gout and possibly reduce healthcare utilization. Additional analyses are needed to confirm that proper sUA management of elderly gout patients reduces healthcare utilization and associated costs. Our results suggest that despite high rates of comorbidities, including renal impairment, the hyperuricemia of elderly gout patients may be more effectively managed with approved doses of febuxostat, with low risk of side effects.

## Conclusions

This analysis of 374 elderly gout subjects demonstrates that ULT with febuxostat 80 mg or 40 mg once daily leads to significantly more subjects achieving a therapeutic goal of sUA <6.0 mg/dL than commonly used doses of allopurinol (200 or 300 mg). ULT was well tolerated despite high rates of comorbidities and concomitant medication use. These results, along with other supporting data, suggest that the underlying hyperuricemia of gout in elderly patients can be more effectively managed with either dose of febuxostat, especially in those patients with mild to moderate renal impairment.

## Abbreviations

ULT: urate-lowering therapies; XO: xanthine oxidase; RCT: randomized controlled trial; sUA: serum urate; eCLcr: estimated creatinine clearance; AE: adverse event; ITT: intent-to-treat

## Competing interests

Pat MacDonald, Barbara Hunt, and Robert L. Jackson are all employees of Takeda Global Research & Development Center, Inc, and were employees of TAP Pharmaceutical Products, Inc, at the time of the study conduct. The study sponsor, Takeda Global Research & Development Center, Inc, was involved in the study design, protocols, subject recruitment, data collections and analyses.

## Authors' contributions

All authors were involved in study concept and design, acquisition of data, data analysis and interpretation, and preparation of the manuscript.

## Pre-publication history

The pre-publication history for this paper can be accessed here:

http://www.biomedcentral.com/1471-2318/12/11/prepub
